# Distribution of *Interferon Lambda 4* Single Nucleotide Polymorphism rs11322783 Genotypes in Patients with COVID-19

**DOI:** 10.3390/microorganisms10020363

**Published:** 2022-02-04

**Authors:** Leonardo Sorrentino, Valentina Silvestri, Giuseppe Oliveto, Mirko Scordio, Federica Frasca, Matteo Fracella, Camilla Bitossi, Alessandra D’Auria, Letizia Santinelli, Lucia Gabriele, Alessandra Pierangeli, Claudio Maria Mastroianni, Gabriella d’Ettorre, Guido Antonelli, Antonio Caruz, Laura Ottini, Carolina Scagnolari

**Affiliations:** 1 Laboratory of Virology, Department of Molecular Medicine, Sapienza University of Rome, 00185 Rome, Italy; leonardo.sorrentino@uniroma1.it (L.S.); giuseppe.oliveto@uniroma1.it (G.O.); mirko.scordio@uniroma1.it (M.S.); federica.frasca@uniroma1.it (F.F.); matteo.fracella@uniroma1.it (M.F.); camilla.bitossi@uniroma1.it (C.B.); dauria.1698419@studenti.uniroma1.it (A.D.); alessandra.pierangeli@uniroma1.it (A.P.); guido.antonelli@uniroma1.it (G.A.); 2 Department of Molecular Medicine, Sapienza University of Rome, 00185 Rome, Italy; valentina.silvestri@uniroma1.it (V.S.); laura.ottini@uniroma1.it (L.O.); 3 Department of Public Health and Infectious Diseases, Sapienza University of Rome, Policlinico Umberto I of Rome, 00185 Rome, Italy; letizia.santinelli@uniroma1.it (L.S.); claudio.mastroianni@uniroma1.it (C.M.M.); gabriella.dettorre@uniroma1.it (G.d.); 4 Department of Oncology and Molecular Medicine, Istituto Superiore di Sanità, 00161 Rome, Italy; lucia.gabriele@iss.it; 5 Immunogenetic Unit, Department of Experimental Biology, University of Jaén, 23071 Jaén, Spain; caruz@ujaen.es

**Keywords:** COVID-19, IFN-Lambda4, single nucleotide polymorphism, rs11322783

## Abstract

Type III interferons (IFN-III), also known as IFN-Lambda, have a pivotal role during SARS-CoV-2 infection. IFN-Lambda response among individuals is heterogeneous and its association with COVID-19 symptoms severity needs to be further clarified. We analyzed the genotype frequencies of *IFNL4* single nucleotide polymorphism (SNP) rs11322783 in patients with COVID-19 (*n* = 128), in comparison with a validated data set of European healthy controls (*n* = 14152). The *IFNL4* SNP was also analyzed according to the haematological and clinical parameters of patients with COVID-19. The distributions of *IFNL4* genotypes among SARS-CoV-2 positive patients [TT/TT 41.4% (*n* = 53), TT/ΔG 47.7% (*n* = 61) and ΔG/ΔG 10.9% (*n* = 14)] and healthy controls were comparable. Different levels of white blood cells (*p* = 0.036) and neutrophils (*p* = 0.042) were found in the *IFNL4* different genotypes in patients with COVID-19; the ΔG/ΔG genotype was more represented in the groups with low white blood cells and neutrophils. There were no differences in major inflammation parameters (C-reactive protein, D-dimer, Albumin, and Lactate-dehydrogenase (LDH)] and survival rate according to the *IFNL4* genotypes. In conclusion, although patients with COVID-19 did not exhibit a different distribution of the *IFNL4* SNP, the ΔG/ΔG genotype was associated with a lower count of immune cell populations. These findings need to be confirmed in larger groups of patients with COVID-19 and the role of *IFNL4* SNP needs to be also investigated in other respiratory viral infections.

## 1. Introduction

The interferon (IFN) response is the first line of defense against pathogens, including respiratory viruses. Two types of IFN are essential to “interfere” with the initial viral replication: type I IFN (IFN-I) and type III IFN (IFN-III), also known as IFN lambda (IFNL) [1,2]. Four IFNL subtypes have been found in humans: IFNL1 (IL-29), IFNL2 (IL-28A), IFNL3 (IL-28B), and IFNL4. IFNL4 shares only ~28% amino acid identity with the other IFNL genes, leading to speculation IFNL4 may have been introduced via a separate duplication event [1,2]. IFNLs are essential components of the mucosal innate immune response, with reported in vitro antiviral activity against respiratory viruses including highly pathogenetic coronaviruses, SARS-CoV-2 and MERS-CoV [1,2,3,4,5].

Genome-wide association studies have linked clearance of hepatitis C virus (HCV) to genetic variations within IFN-III loci [6], and this subsequently led to the discovery of the *IFNL4* gene [7] Studies indicate that *IFNL4* protein can induce IFN-stimulated genes (ISGs) through activation of the Janus kinase (JAK)-signal transducer and activator of transcription (STAT) pathway and exert antiviral effects [5,7]. Upon identification of the *IFNL4* gene, Prokunina-Olsson et al. found a dinucleotide genetic variant (TT/TT, TT/ΔG or ΔG/ΔG), situated in the first exon of this gene [7]. Only individuals carrying the ancestral *IFNL4*-ΔG allele of the single nucleotide polymorphism (SNP) named rs368234815, now merged in rs11322783 [8], are able to synthesize the full-length functional *IFNL4* protein [9]. By contrast, *IFNL4* TT, leads to a frameshift and therefore to aborted expression of *IFNL4* protein [7]; the allele frequency for IFNL4-ΔG varies markedly by population (with a frequency of 95% in Africa, around 50% in Europe, and 15% in Asia) and seems to be under negative selection [7]. The rs11322783 SNP is in strong linkage disequilibrium with the genetic variation, SNP rs12979860, located within intron 1 of *IFNL4* and associated with spontaneous and IFN therapy-induced HCV clearance [7,10,11,12,13,14,15,16]. In particular, carriers of the CC genotype at rs12979860 or of the TT genotype at rs11322783 are more likely to spontaneously clear acute HCV infection or to better respond to IFN treatments than individuals with rs12979860 T or rs11322783 ΔG allele [7,10,11,12,13,14,15,16]. Given that the better response to HCV is probably not the evolutionary driver against the expression of *IFNL4* protein [2], many studies in other infectious diseases have been conducted, but only a few reported associations with variants in the IFNL3 and IFNL4 region. Both the unfavourable rs11322783 ΔG and rs12979860 T alleles were associated with impaired clearance of other RNA viruses, including Rhinovirus and Enterovirus [17]. In partial disagreement, our previous study in children bronchiolitis cases indicate that rs12979860 and rs8099917 SNPs had no impact on the clinical course of bronchiolitis with the only exception of the rs12979860 TT genotype which increased the risk of hospitalization for bronchiolitis at an earlier age [18].

As far as the impact of the *IFNL4* SNPs in SARS-CoV-2 infection is concerned, Agwa et al. observed that the CC genotype in *IFNL4* SNP rs12979860 is more frequent in patients with COVID-19 than in healthy controls in Egypt [19]. On the contrary, Saponi-Cortes et al. found that the rs12979860 T allele was associated with COVID-19 incidence in Spain [20]. Additionally, patients who simultaneously expressed *IFNL4* SNP (rs11322783 TT/TT) and genotypes of other SNPs (rs12979860 CC, rs12980275 AA, rs8099917 TT) were associated to survivability to SARS-CoV-2 infection [19]. Moreover, SNP rs1297860 TT genotype and SNP rs11322783 ∆G/∆G genotype in patients with COVID-19 indicated a lower ability in SARS-CoV-2 clearance [21].

Remarkably, heterogeneous IFN-III responses in relationship with severity of COVID-19 has been observed. Multiple studies found that reduced expression levels of IFN-III were associated with patients’ worse outcomes, reducing SARS-CoV-2 clearance [22]. Our previous study showed a general decreased expression of IFNL1-3, IFN-I, and ISGs-mRNAs in critically ill patients with COVID-19 that required invasive mechanical ventilation [23]. In agreement, Sposito et al. reported that IFNL1, IFNL3, and ISGs expression is lower in patients with severe COVID-19 [24]. Moreover, a negative correlation between IFNL2 gene expression levels and severity of symptoms has been shown [19]. In this regard, the presence of *IFNL4* SNPs has been associated with alterations in the expression level of IFN-III and ISGs [25,26,27]: subjects carrying CC genotype in rs1297860 SNP had a higher expression of ISGs, although T allele was associated with increased expression of IFNα and IFNβ [25]. However, data remains conflicting; indeed, ISGs expression is not influenced by the presence of rs11322783 ΔG allele in HIV-1 infected patients [28]. More recently, Azim Ansari et al. found that expression of Angiotensin-converting enzyme 2 (ACE2), the functional receptor for SARS-CoV-2 entry into host target cells, is negatively correlated with *IFNL4* production [29]. Remarkably, ACE2 transcripts’ level (all isoforms) in vivo were correlated with those of ISG15, a marker of type I and III IFNs’ activation in patients suffering from respiratory diseases not caused by SARS-CoV-2 [30].

Thus, in order to provide insight into the impact of the *IFNL4* SNPs in SARS-CoV-2 infections, we investigated whether genotypes of rs11322783 SNP were differently distributed between patients with COVID-19 and the European validated group of healthy controls [31]. We also examined whether the presence of the ΔG allele was associated with demographic and clinical data, as well as with the rate of intensive care unit (ICU) admission, and severe outcome of COVID-19.

## 2. Methods

### 2.1. Study Group

A total of 128 Caucasian patients (≥18 years) were recruited at the Division of Infectious Diseases, Department of Public Health and Infectious Diseases, Hospital of Sapienza University of Rome (Italy) with laboratory-confirmed SARS-CoV-2 infection. Nasopharyngeal swabs were collected within 48 hours of hospital admission for SARS-CoV-2 detection. Our study was delineated by eligibility criteria shared by all enrolled participants. Inclusion criteria were as follows: (i) all individuals who provided informed consent prior to the start of study procedure; (ii) male and female adults ≥ 18 years of age; (iii) diagnosis of SARS-CoV-2 at the day of hospital admission. Exclusion criteria were as follows: to have not signed the informed consent; pregnancy status, human immunodeficiency virus (HIV) infection, contraindications for taking blood samples.

All the hospitalized patients received therapeutic regimens including dexamethasone (6 mg a day), low molecular weight heparin for prophylaxis of deep vein thrombosis as recommended at the time by the Italian Society of Infectious Diseases [32] and standard of care treatments. Amongst all participants, demographic and clinical data were obtained from electronic medical records in the Hospital Electronic Information System. Variables considered for the study included: age, gender, admission and discharge date from the hospital, length of hospitalization; cardiovascular (CV) disease, haematological and inflammation parameters [count of blood immune cell, C-reactive protein, D-dimer, Albumin, Lactate-dehydrogenase (LDH)], thrombotic events, blood bacterial infection and bacterial pulmonary superinfection. A predictive model, Comorbidity, Age, Lymphocyte count and Lactate dehydrogenase (CALL), has been devised to estimate progression towards severe forms of COVID-19 with optimal sensitivity and specificity. [33]. The CALL score ranges from 4 (absence of comorbidity, age under 60 years, lymphocyte count over 1.0 × 10^9^/L, LDH under 250 U/L) to 13 (presence of comorbidity, age over 60 years, lymphocyte count under 1.0 × 10^9^/L, LDH over 500 U/L). Blood samples were collected from each patient during the hospitalization. The study was approved by the institutional review board (Ethics Committee of Umberto I General Hospital Rif. 5836, Prot. 0690/2021). All study participants gave written informed consent.

### 2.2. IFNL4 Genotyping

Viral RNA was extracted from nasopharyngeal swabs using the Versant SP 1.0 Kit (Siemens Healthcare Diagnostics, Milan, Italy). In particular, 10 µl of extracted RNA was reverse-transcribed and simultaneously amplified using a real-time RT-PCR system (RealStar SARS-CoV-2 RT-PCR, Altona Diagnostics, Hamburg, Germany), targeting E and S genes of SARS-CoV-2. TaqMan probe specific for the E gene is labeled with FAM reporter, while TaqMan probe specific for the S gene is labeled with Cy5 reporter, as previously described [32]. Then, SNP genotyping was carried out on purified whole nucleic acids from blood samples (DNeasy Blood and Tissue Kit, QIAGEN, Milan, Italy) collected from all SARS-CoV-2 positive patients. Briefly, 100 µL of blood sample were mixed with 20 µL of Proteinase K and 100 µL of PBS. Then, 200 µL of AL buffer were added and incubated at 56 °C for 10’ minutes. After the incubation, 200 µL of 96% ethanol were added, and transferred to the spin column. A centrifugation at 8000 rpm for 1 minute was performed and the flow-through was discarded. Next, 500 µL of AW1 buffer were added and after a centrifugation at 8000 rpm for 1 minute; the flow-through was discarded and the same step was repeated with AW2 buffer performing a centrifugation for 2 minutes. Lastly, each column was eluted in 200 µL of AE buffer. Genotyping allelic discrimination was performed by the TaqMan method (StepOne Plus Real-Time PCR System, A.B. Foster City, CA, USA) using specific primers for the amplification of the polymorphic sequence and two TaqMan-MGB probes (VIC and FAM) specific for each allele (Express program and Genotyping assay service Applied Biosystem) as previously reported [28]. In particular, TaqMan probe with FAM dye label recognized the wild type allele (TT) while the TaqMan probe with VIC dye label was complementary to the variant allele (ΔG). For one well, 5 μL of individuals DNAs were added to a mixture of final volume of 15 μL containing 10 μL 2x Probes Master Mix, 1 μL of SNP mixture and 4 μL of nuclease-free water. Allelic discrimination was evaluated according to the variation of reported dye fluorescence signals among genotypes clusters.

### 2.3. Data Analysis

Genotyping was conducted in a blinded fashion relative to patient characteristics. Before testing for SNP, samples were anonymized, and a unique randomly generated identification code was assigned to each record and the correspondent swab. Researchers performing genetic analyses were unable to identify patients at all stages, and no permanent record linking these data to patient IDs was produced. For the present study, we relied on genotype frequencies of *IFNL4* single-nucleotide polymorphism rs11322783 from a validated data set of European healthy subjects (*n* = 14152) [31]. All data were analyzed, and graphs were generated using STATA software, version 17.0 (StataCorp LCC, College Station, TX, USA). All measurements were expressed as median (Range). The demographic and clinical characteristics of SARS-CoV-2-infected patients and healthy controls were compared using the Chi-squared test and Mann Whitney U test. Survivability analyses were performed according to Kaplan–Meier method and univariate Cox regression model. Tests for deviation from Hardy-Weinberg equilibrium and Armitage’s trend tests were used to evaluate deviation between observed and expected frequencies for identification of unexpected population or genotyping biases in genetic frequencies of rs11322783 SNP in the patients with COVID-19. A logistic regression model was used to determine the allele and genotypes distribution in patients stratified by white blood cells (WBC) and neutrophils groups (high, medium, low). A *p*-value below 0.05 was considered significant.

## 3. Results

### 3.1. Clinical Features of SARS-CoV-2 Infected Patients

We enrolled SARS-CoV-2-infected patients (*n* = 128), of which 49 (38.3%) were female, with a median age of 64 years. Demographic and clinical features of patients with COVID-19 are shown in Table 1. The median length of hospitalization was 19 days (Range: 1–86). Amongst SARS-CoV-2-infected individuals, 24 (18.7%) required Intensive Care Unit (ICU) admission because of pulmonary embolism. Moreover, 15 (11.7%) cases had thrombotic events, 13 (10.2%) had a bacterial blood infection and 12 (9.4%) cases had bacterial pulmonary superinfection. Patients were stratified in three groups according to the CALL clinical score resulting in: 29 (22.6%) with low CALL (4–6), 45 (35.2%) with intermediate CALL (7–9) and 54 (42.2%) with high CALL (10–13) severity score.

### 3.2. IFNL4 SNPs in Patients with COVID-19

The frequencies of *IFNL4* rs11322783 genotypes in the patients with COVID-19 (*n* = 128) and a validated data set of European healthy controls (*n* = 14152) [31] were as follows: TT/TT 41.4% (*n* = 53) vs. 45%, TT/ΔG 47.7% (*n* = 61) vs. 44.2% and ΔG/ΔG 10.9% (*n* = 14) vs. 10.8%; there was no statistically significant difference in the distribution of *IFNL4* genotypes (*p* > 0.05 for all the measurements). Then, we evaluated whether the distribution of the *IFNL4* rs11322783 genotypes in patients with COVID-19 varied according to the count of blood immune cells (total white blood cells number, neutrophils, lymphocytes, monocytes, platelets), levels of inflammation parameters (C-reactive protein, D-dimer, Albumin and LDH), and the rate of ICU admission, thrombotic events, blood bacterial infection (caused by *E. coli*, *S. epidermidis, E. faecalis, S. aureus, A. baumannii* and *S. hominis*), and bacterial pulmonary superinfection (caused by *P. aeruginosa* and *K. pneumoniae*). Different levels of white blood cells (WBC) (*p* = 0.036) and neutrophils (*p* = 0.042) were found in patients with COVID-19 among the three different genotypes (Table 2). In addition, the logistic regression model used to determine the allele distribution in the groups (medium, high, low), showed that the ΔG/ΔG genotype was significantly more represented in those with lower WBC and neutrophils (Table 3).

No other statistically significant relationships were observed for the remaining studied variables concerning the *IFNL4* genotypes.

### 3.3. Survival Analysis in Patients with COVID-19

**Figure 1:**
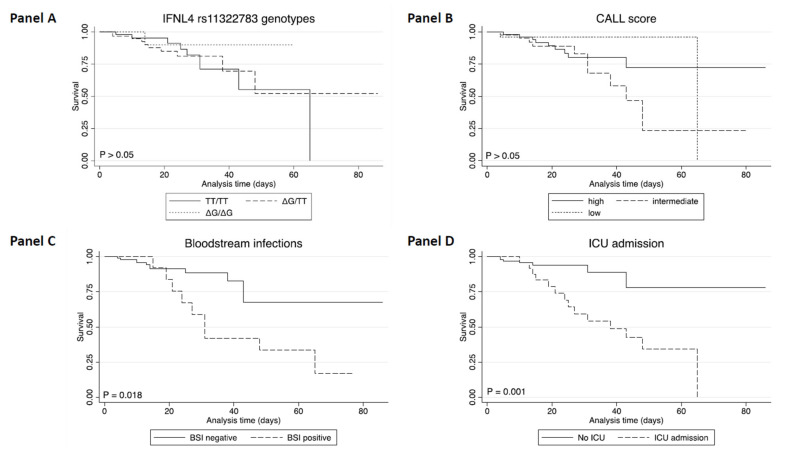
Survival rate according to IFNL4 rs11322783 SNP and clinical parameters in patients with COVID-19 by Kaplan–Meier Plotter. Survival Kaplan–Meier curves according with TT/TT, ∆G/TT and ∆G/∆G genotypes (*p* > 0.05, Panel (**A**)), CALL score categories (*p* > 0.05, Panel (**B**)), the presence of BSI (*p* = 0.018, Panel (**C**)), and ICU admission (*p* = 0.001, Panel (**D**)).

Survival analysis showed that *IFNL4* rs11322783 genotype distribution was not associated with patients’ outcome (Figure 1, Panel A). Moreover, survival analysis revealed no significant differences according to the age, gender, and the CALL score assigned to each COVID-19 patient (Figure 1, Panel B). As expected, those patients transferred to ICU (*p* = 0.001) or with bloodstream infections (BSI, *p* = 0.018) had a worse overall survival outcome compared to those not admitted to ICU and without BSI (Figure 1, Panel C and D).

## 4. Discussion

IFN-III represents the most recently discovered members of the IFN system. Similar to IFN-I, IFN-Ls participate in defense against viruses mostly on cells of epithelial origin such as bronchial epithelium, gastrointestinal epithelium, and keratinocytes, according to the limited tropism of IFN-III receptor [1,2]. Moreover, as proof of the key role of IFN-III in the regulation of antiviral immunity, SNPs in IFNL4 loci were associated with clinical outcomes of different viral infections including those caused by SARS-CoV-2 [17,19,21].

In this study, we analyzed the distribution of the *IFNL4* rs11322783 SNP in hospitalized SARS-CoV-2 infected patients. Our results indicate that rs11322783 *IFNL4* genotypes are similarly distributed between patients with COVID-19 and healthy controls. Moreover, the *IFNL4* SNP seems to not influence the clinical outcome of COVID-19 being not related to clinical severity (CALL score or ICU admission) and patients’ survival outcomes. IFNLs are known to play a pivotal function during respiratory infections, including those caused by Respiratory Syncytial Virus (RSV) [34], Influenza A [35], and also SARS-CoV-2 [36]. IFN-III proper activation may control SARS-CoV-2 replication, promoting virus clearance and impairing progression to severe forms of COVID-19 [36]; an heterogenous response has been documented in patients with COVID-19, according to their clinical status [22,23]. The *IFNL4* producing ΔG/ΔG genotype has been associated with higher viral loads in patients with COVID-19 [37], but its contribution remains controversial [19,20]. Indeed, individuals who carry CC genotype of SNP rs12979860 showed a higher incidence of COVID-19 compared to the others [19]. On the other hand, the T allele of rs12979860 was overrepresented in patients with COVID-19 with regard to the general healthy population, indicating that this allele could be a risk factor for COVID-19 [20]. In this context, the CC genotype (rs12979860) was significantly lower in patients with COVID-19 compared to healthy controls [20], underlining the increasing complexity of this immunoregulatory network.

In this study, we found different levels of total WBC and neutrophils among *IFNL4* SNP genotypes; in particular, patients with COVID-19 homozygous for the *IFNL4*-producing allele were more represented in the group with low-counts of WBC and neutrophils.

Lymphopenia and higher levels of neutrophils and WBC have been frequently reported as markers of disease severity and mortality in COVID-19 [33]. Moreover, it is known that during early phases of viral infection, neutrophils are recruited from the circulation into the infectious site, and promote oxidative damage, phagocytosis, and virus clearance [38]. In this regard, an additional antimicrobial/antiviral function of neutrophils relies on a special type of programmed cell death called neutrophil extracellular traps (NETs) formation [39]. These NETs correspond to extracellular filaments of uncondensed chromatin (an association of DNA and histones) covered by numerous proteins of mainly granular origin [40]. An abnormal formation of NETs has been observed during severe COVID-19 and has been shown to contribute to lung damage and worse outcome of SARS-CoV-2 infection [41]. Increased plasma NET levels were observed in non-intubated patients with COVID-19, as well as in endotracheally intubated patients with COVID-19, compared with healthy donors and convalescent patients [42,43]. Accordingly, it has been demonstrated that SARS-CoV-2 is able to activate NETosis and increase levels of intracellular Reactive Oxygen Species (ROS) in human neutrophils [44]. In this study, patients with ΔG/ΔG genotype presented lower levels of neutrophils and white blood cells that could influence the outcome of COVID-19. Indeed, during the early phase of SARS-CoV-2 infection, innate immunity, including that associated with IFN-I [45] and III [46] response, might control the viral spreading, limiting the rate of SARS-CoV-2 replication in the upper respiratory tract. Moreover, COVID-19 severe forms are characterized by the so-called “cytokine storm” and increased levels of neutrophils [47,48]. Formation of NETs can induce the production of IL1β by macrophages, and, simultaneously, IL1β can induce NETs formation [49] generating an IL1β-NETs loop. At the same time, neutrophils express stable IFN-L receptor (IFNLR) [50] and produce ISGs during bacterial and viral infections [51,52]. It has been reported that deregulated expression of ISGs in neutrophils during acute respiratory distress syndrome (ARDS) is associated with worst outcome [51]. However, mice treated with IFN-L had reduced migratory capacity of neutrophils in tissues. Indeed, it has been shown that IFN-L treatment reduces neutrophils infiltration in arthritis [50] and these data was further confirmed in mice with the observation of a lower migration of neutrophils in gut during autoimmune diseases after treatment with IFN-L [53]. However, it remains unclear whether *IFNL4* can regulate neutrophil response and its impact on neutrophil functions and related IFN-III pathways in COVID-19 individuals. Indeed, we did not find any differences in TT/ΔG and TT/TT frequencies based on neutrophils and WBC levels (Table 3). Moreover, genome-wide association studies (GWAS) did not find any correlations between IFNL4 SNP and COVID-19 [54,55,56], suggesting that IFNL4 SNP might have a small or no impact during SARS-CoV-2 infection. The latter aspect needs to be further investigated in a higher number of patients with COVID-19; indeed, a previous study [21] found that the ΔG/ΔG genotype, in an Iranian population suffering from COVID-19, is associated with low survivability. Our results suggest that the role of *IFNL4* in respiratory viral infections, including that caused by SARS-CoV-2, deserves to be better characterized.

## Figures and Tables

**Table 1:** Demographic, clinical, and biochemical features of SARS-CoV-2 infected patients.

Features	COVID-19 Patients (*n* = 128)
Age at diagnosis (years) (mean (range))	63.9 (25–95)
Gender (N (percentage))	
Male	79 (61.7)
Female	49 (38.3)
CALL score (N (percentage))	
Low severity (4–6)	29 (22.6)
Intermediate severity (7–9)	45 (35.2)
High severity (10–13)	54 (42.2)
Clinical features (N (percentage))	
ICU	24 (18.7)
Thrombotic events	15 (11.7)
Death	21 (16.4)
BSI	13 (10.2)
Bacterial pulmonary superinfection	12 (9.4)
Blood parameters (mean (range))	
WBC cell/mm^3^	6293.6 (2110–19150)
Neutrophils cell/mm^3^	4691.2 (1120–18000)
Lymphocytes cell/mm^3^	1067.7 (110–4760)
Monocytes cell/mm^3^	361.7 (150–1040)
CRP µg/L	98380 (300–540000)
D-dimer µg/L	1690 (176–4610)
Albumin g/L	36.9 (19–46)
LDH U/L	335 (111–1249)
Platelets cell/mm^3^	221 × 10^3^ (65–516)

ICU: intensive care unit; BSI: bloodstream infections; WBC: white blood cells; CRP: C-reactive protein; LDH: lactate dehydrogenase.

**Table 2:** Correlation between IFNL4 genotypes with counts blood immune cells, levels of inflammation parameters, and COVID-19 outcomes.

Features	Ranges *	*IFNL4* SNP TT/TT	*IFNL4* SNP ΔG/TT	*IFNL4* SNP ΔG/ΔG	*p*-Value
SARS-CoV-2 patients		53 (41.4)	61 (47.7)	14 (10.9)	
WBC cell/mm^3^	4.5–11.0 × 10^3^	40 (75.5)	52 (86.7)	7 (50.0)	**0.036**
	<4.5 × 10^3^	7 (13.2)	5 (8.3)	5 (35.7)	
	>11.0 × 10^3^	6 (11.3)	3 (5.0)	2 (14.3)	
Neutrophils cell/mm^3^	1.5–8.0 × 10^3^	43 (81.1)	52 (86.6)	8 (57.1)	**0.042**
	<1.5 × 10^3^	3 (5.7)	4 (6.7)	4 (28.6)	
	>8.0 × 10^3^	7 (13.2)	4 (6.7)	2 (14.3)	
Lymphocytes cell/mm^3^	1.0–4.0 × 10^3^	43 (81.1)	50 (83.3)	10 (71.4)	0.59
	<1.0 × 10^3^	10 (18.9)	10 (16.7)	4 (28.6)	
	>4.0 × 10^3^	-	-	-	
Monocytes cell/mm^3^	0.1–0.7 × 10^3^	49 (92.4)	60 (98.4)	14 (100)	0.47
	<0.1 × 10^3^	3 (5.7)	1 (1.6)	0 (0.0)	
	>0.7 × 10^3^	1 (1.9)	0 (0.0)	0 (0.0)	
CRP µg/L	<8.0 × 10^3^	8 (15.1)	6 (9.8)	3 (21.4)	0.45
	>8.0 × 10^3^	45 (84.9)	55 (90.2)	11 (78.6)	
D-dimer µg/L	<500	5 (11.4)	9 (16.4)	2 (14.3)	0.78
	>500	39 (88.6)	46 (83.6)	12 (85.7)	
Albumin g/L	35–55	30 (66.7)	30 (53.6)	8 (61.5)	0.41
	<35	15 (33.3)	26 (46.4)	5 (38.5)	
	>55	-	-	-	
LDH U/L	80–300 × 10^3^	16 (38.8)	21 (35.0)	4 (28.6)	0.47
	<80 × 10^3^	4 (7.7)	1 (1.7)	0 (0.0)	
	>300 × 10^3^	32 (61.5)	38 (63.3)	10 (71.4)	
Platelets cell/mm^3^	150–450 × 10^3^	44 (83.0)	45 (76.3)	12 (85.7)	0.8
	<150 × 10^3^	7 (13.2)	12 (20.3)	2 (14.3)	
	>450 × 10^3^	2 (3.8)	2 (3.4)	0 (0.0)	
Call	Low severity (4–6)	13 (24.6)	13 (21.3)	3 (21.4)	0.94
	Intermediate severity (7–9)	20 (37.7)	20 (32.8)	5 (35.7)	
	High severity (10–13)	20 (37.7)	28 (45.9)	6 (42.9)	
ICU admission rate	yes	11 (20.7)	11 (18.0)	2 (14.3)	0.84
	no	42 (79.3)	50 (82.0)	12 (85.7)	
Thrombotic events	Positive	5 (9.4)	8 (13.3)	2 (14.3)	0.78
	Negative	48 (90.6)	52 (86.7)	12 (85.7)	
Bloodstream infections (BSI)	Positive	6 (11.8)	6 (10.7)	1 (7.7)	0.91
	Negative	45 (88.2)	50 (89.3)	13 (92.3)	
Bacterial pulmonary superinfections	Positive	6 (12.2)	4 (7.3)	2 (15.4)	0.57
	Negative	43 (87.8)	51 (92.7)	11 (84.6)	

Data are represented as total numbers (percentage) of SARS-CoV-2 patients grouped by IFNL4 genotypes. Statistical analyses were performed using Chi square test. In bold are represented significative *p*-values. * For WBC, neutrophils, lymphocytes, monocytes, albumin, LDH and platelets, normal, low and high levels are shown respectively. For CRP and D-dimer, normal and high levels are shown, respectively.

**Table 3:** Differences in *IFNL4* allele and genotype frequencies among normal and abnormal ranges of WBC and neutrophils in SARS-CoV-2 patients.

			Allele Frequencies Comparison	Heterozygous and Homozygous Comparison	Homozygous and Homozygous Comparison	Allele Positivity Comparison	Armitage’s Trend Test
WBC	Normal levels vs. low levels	allele T	0.22	0.33	0.04	0.95	0.20
		allele ∆G	0.22	0.003	0.04	0.005	0.20
	Normal levels vs. high levels	allele T	0.89	0.18	0.48	0.37	0.88
		allele ∆G	0.89	0.08	0.48	0.20	0.88
Neutrophils	Normal levels vs. low levels	allele T	0.04	0.90	0.01	0.35	0.04
		allele ∆G	0.04	0.01	0.01	0.003	0.04
	Normal levels vs. high levels	allele T	0.82	0.25	0.63	0.41	0.81
		allele ∆G	0.82	0.19	0.63	0.36	0.81

Data are shown as *p*-values calculated with test for association. Test for deviation from Hardy–Weinberg equilibrium (data not shown) showed no significative differences (*p* > 0.05). WBC: white blood cells.

## Data Availability

Not applicable.
